# Contextual influences on sentence repetition as a tool for the identification of language impairment in Grade 3 Sepedi-English bilinguals: A case against bilingual norms

**DOI:** 10.4102/sajcd.v67i1.762

**Published:** 2020-09-04

**Authors:** Heila Jordaan, Monene H. Ngwanduli

**Affiliations:** 1 Speech Pathology and Audiology, Faculty of Humanities, University of the Witwatersrand, Johannesburg, South Africa

**Keywords:** sentence repetition, Sepedi-English bilingual, specific language impairment, educational contexts

## Abstract

**Background:**

Specific language impairment (SLI) is difficult to identify because it is a subtle linguistic difficulty, and there are a few measures available to differentiate between typical and atypical language development in bilinguals. Sentence repetition (SR) has strong theoretical foundations and research evidence as a valid tool for the identification of SLI in bilinguals.

**Objective:**

This study assessed the value of SR using peer group comparisons to identify Sepedi-English bilingual children at the risk of SLI.

**Method:**

One hundred and two Grade 3 learners in three different contexts of education were assessed on equivalent English and Sepedi SR measures.

**Results:**

Eleven participants who scored between 1 and 2 standard deviations (SD) below the peer group means on both the English and Sepedi SR tests were identified with possible SLI. Learners in the English language of learning and teaching (LoLT) – Sepedi additional language (SAL) context obtained similar scores in both languages, a higher score in English than the English LoLT group and a higher score in Sepedi than the Sepedi LoLT – EAL group. The English LoLT group obtained a significantly higher score in English than in Sepedi and a significantly lower score than the other two groups in Sepedi. The Sepedi LoLT group obtained a significantly higher score in Sepedi than in English, their additional language, in which they obtained a significantly lower score than the other two groups.

**Conclusion:**

Sentence repetition tasks are valid screening tools to identify bilingual children with SLI by comparing them to peer groups. The SR tests were sensitive to language practices in different educational contexts. It was observed that a bilingual approach that uses both English and the home language as academic languages leads to better language outcomes.

## Introduction

South African speech-language therapists are tasked with the identification of language impairment in children who speak a variety of languages and, in most cases, a combination of languages, because the majority of the population are bilingual or multilingual.

Specific or primary language impairment (SLI) is particularly difficult to identify because it occurs in the absence of any obvious physical, cognitive, neurological, visual or hearing disabilities (Bishop, Snowling, Thompson, Greenhalgh, & the CATALISE-2 Consortium, 2017). The term SLI describes a subset of children with developmental language disorder (DLD) who, when compared to their typically developing peers, demonstrate an impairment in language that is not based on a biomedical condition or related to an intellectual difficulty (Volkers, 2018). A group of child language clinicians and researchers across the world (the CATALISE consortium) used the Delphi process to arrive at a consensus definition for the broader term, DLD. This group recommended that the term DLD be used to describe children who have an impairment in language, with or without an intellectual disability (Green, 2020), thus contrasting the term with SLI.

The manifestations of SLI are linguistic and often subtle, so it may go undetected in the preschool period (Tomblin, Nippold, Fey, & Zhang, 2014), particularly in the South African context, where basic physical, health and nutrition needs take preference over early language development. When children with SLI start formal schooling, their language problems may affect their literacy and academic skills, and their communication impairments may be addressed for the first time.

Bilingual children with SLI will demonstrate impairments in both languages and should therefore be assessed in both languages (Kohnert, 2010). However, the identification of SLI in bilingual children is complicated by similarity in the language characteristics of typically developing bilingual children and children with SLI (Armon-Lotem & De Jong, 2015) as well as variations in the linguistic skills within and across the languages of bilingual children (Kohnert, 2010). The lack of appropriate assessment tools for bilingual children adds to this dilemma and may result in over- or under-identification of language impairment (Bedore & Pena, 2008; Restrepo & Guti´errez-Clellen, 2004). The consequences are that bilingual children are either over-represented in special educational services or do not receive intervention at all because their difficulties are attributed to acquiring two language systems.

In this study, we propose that sentence repetition (SR) may be a valuable screening tool in the identification of SLI in bilingual children because it has a strong theoretical basis and supporting research evidence.

Historically, SR tasks have long been included in test batteries for language, for example, the Clinical Evaluation of Language Fundamentals (CELF)–R (Semel, Wiig, & Secord, 1994) and the Test of Language Development–Primary (TOLD–P) (Newcomer & Hammill, 1997) or general abilities, for example, Wechsler Preschool and Primary Scale of Intelligence – Revised (Wechsler, 1992). Sentence repetition tasks have also been used to explore the processing abilities of children with SLI and other conditions (e.g. Ellis-Weismer, Evans, & Hesketh, 1999; Redmond, 2005). In the early 2000s, studies began to explore the use of SR as a clinical marker for SLI (e.g. Conti-Ramsden, Botting, & Faragher, 2001).

Currently, there is substantial research evidence for the sensitivity and specificity of SR tasks in the identification of SLI in monolingual children who speak English (Conti-Ramsden et al., 2001; Oetting, McDonald, Seidel, & Hegarty, 2015) as well as other languages (Christensen, 2019; Leclercq, Qu´emart, Magis, & Maillart, 2014; Stokes, Wong, Fletcher, & Leonard, 2006). Recently, there have also been studies to support SR as a means of distinguishing between typical and atypical language development in bilingual children (Armon-Lotem & Meir, 2016; Ebert, 2014; Fitton, Hoge, Petscher, & Wood, 2019; Hamann & Abed Ibrahim, 2017; Thordardottir & Brandeker, 2013).

Theoretically, the multicomponent working memory model (Baddeley, 2012) views SR tasks as depending primarily on the efficiency of an episodic buffer (Alloway, Gathercole, Willis, & Adams, 2004), which links the subsystems of working memory and long-term memory (Repovs & Baddeley, 2006) and is believed to place constraints on language acquisition (Boyle, Lindell, & Kidd, 2013). In addition, in line with the regeneration hypothesis proposed by Lombardi and Potter (1992), SR is a multi-faceted task that engages virtually all aspects of language processing. After hearing a sentence, the listener creates a conceptual representation of the sentence and then goes through a series of processes including activating relevant lexical knowledge, grammatical encoding, phonological realisation and speech production to repeat the sentence (Klem et al., 2015).

The methods employed in the studies mentioned above, involve using SR to assess children already identified with SLI and comparing them to control groups of typically developing peers. In this study, we adopt a different approach to assess the value of SR in identifying children with SLI by comparing them to peers who speak the same language pair and have had the same experiences with their exposure to the two languages. We argue that even bilingual children who speak the same language pairs are not a homogenous group. In their study, Armon-Lotem and Meir (2016, p. 716) proposed that bilingual children should be compared to ‘bilingual norms’ as opposed to ‘monolingual norms’ to improve the diagnostic accuracy of SR. Whilst we agree with the strategy of comparing bilingual children to age-matched peers, we hypothesise that SR accuracy will depend on exposure to and experience of the learners in each language.

Therefore, in this study, we assessed three groups of bilingual children in different contexts of education, where the use of their two languages varied considerably. These contexts have evolved out of the post-apartheid education system in which language-in-education practices should promote additive bilingualism, which enables all the learners to acquire English, the language of social, economic and educational advancement, whilst still maintaining the home language (Language in Education Policy, 1997). In the first context, the language of learning and teaching (LoLT) is English from Grade 1, whilst the children still speak their home language (Sepedi) at home.

In the second context, the LoLT is the home language (Sepedi) until Grade 4 and English is taught as an additional language in the foundation phase. We were also able to identify a third context in which the LoLT is English and Sepedi is taught as an additional language.

Sepedi is the standardised dialect of Northern Sotho (Sesotho sa Leboa) and is one of the 11 official languages in South Africa. According to the 2011 census, it was the first language of 4 618 576 people, principally in the provinces of Limpopo, Gauteng and Mpumalanga (Posel & Zeller, 2016).

## Aim

The aim of the study was to evaluate SR in English and Sepedi as a tool to identify bilingual children with SLI in three educational contexts.

## Objectives

To compare the SR scores obtained by the participants in each of the three educational contexts. To compare the SR scores obtained by the participants in English and Sepedi. To identify children within each educational context who may present with SLI.

## Research methods and design

### Study design

The design of this study was quantitative, comparative and cross-sectional in nature (Schiavetti, Metz, & Orlihoff, 2011). The study was quantitative in that the SR assessments in English and Sepedi yielded numerical data, which were analysed using statistical methods. The study comprised both within (participants’ scores on the English and Sepedi SR tests) and between group comparisons (SR scores in both English and Sepedi were compared between the three contexts). The study was cross-sectional as the data was collected at a single point in time. There was no experimental manipulation of variables.

## Setting

The study took place at three schools located in the Seshego and Polokwane areas of the Limpopo Province.

The three schools represented the three educational contexts described in the introduction. The first (English LoLT) and the third (English LoLT- Sepedi additional language) contexts were suburban public primary schools across the street from each other in the eastern part of Polokwane. The first was an ex-model-C school (Webb, Lafon, & Pare, 2010) and the third was a school that was opened 2 years post-democracy in 1996. Mostly, children from middle class backgrounds attended both the schools. The second context (Sepedi LoLT - English additional language) was a township school in central Seshego, attended by children of mostly poor backgrounds.

## Study population and sampling strategy

A total of 102 Grade 3 learners with an average age of 8 years, 8 months participated in the study. Table 1 provides a description of the participants in each context in terms of number, gender and mean age.

**TABLE 1:** Description of participants.

Variable	Context		
	1: English LoLT (ELoLT)	2: Sepedi LoLT ‒ English additional language (SLoLT-EAL)	3: English LoLT ‒ Sepedi additional language (ELoLT-SAL)
Number of participants	36	35	31
**Gender**			
Female	21	21	17
Male	15	14	14
Mean age	8 years 9 months	8 years 7 months	8 years 7 months

LoLT, language of learning and teaching; ELoLT, English LoLT; EAL, English additional language; SAL, Sepedi additional language.

Non-probability, purposive sampling was employed to select participants who met the selection criteria. The principals of the identified primary schools were approached to allow learners to participate in the study.

Information sheets, consent forms and parent questionnaires were distributed to the parents and/or legal guardians of all Grade 3 learners at each school, and those learners whose parents and/or legal guardians gave written permission for their participation were included in the study provided they met the selection criteria.

## Criteria for selection of participants

Learners were required to be in Grade 3 of their respective schools to ensure that they had sufficient experience with the respective languages to cope with the SR tasks. Previous research (Marshall, 2013) showed that Grade 3 learners coped better with SR tasks than Grade 1 and 2 learners. They were also required, according to parental questionnaire, to speak Sepedi as their primary home language and to comply with the formal definition of sequential bilingualism, that is, to have acquired English as an additional language after the age of three (Genesee & Nicoladis, 2009) to ensure homogeneity with respect to bilingual acquisition profile.

Learners should not have suspected, reported or identified physical, cognitive, hearing or visual impairments that may have obviously affected their language development. However, learners with subtle language impairments were included as the study aimed to identify them.

## Research instrumentation

### Parent questionnaire

A parent of each learner was requested to complete a questionnaire to obtain information pertaining to the dominant home language and other languages spoken, relative amount of exposure to English and other languages at home, years of exposure to English, milestones in the development of the home language and general health and development.

### Recalling sentences subtest from the clinical evaluation of language fundamentals-4 (CELF-4)

The recalling sentences subtest of the CELF, 4th edition (CELF-4) (Semel, Wiig, & Secord, 2003) was used as the English SR measure. This subtest, consisting of 32 items, assesses the ability to recall sentences of increasing length and complexity and repeat them without changing word meanings or sentence structure.

This SR test was used in this study because previous local research had shown that it was a valid test for use with English language learners. A study by Marshall (2013) found a strong positive correlation between the recalling sentences subtest (Semel et al., 2003) and the Redmond sentence repetition test (Redmond, 2005) [*r* = 0.69; *p* > *r* = 0.001), which establishes concurrent validity. In turn, the Redmond sentence repetition test (Redmond, 2005) was found to have a strong correlation with all language measures on the diagnostic evaluation of language variation-criterion referenced test (DELV-CR) (Seymour, Roeper, & de Villiers, 2003) in a study by Jordaan (2011). This supports the research evidence suggesting that SR is related to virtually all aspects of language processing (Klem et al., 2015). The use of the DELV-CR as an accurate measure of language processing in South African children has been piloted in a number of studies (Jordaan, 2011; Marshall, 2013; Meirim, Jordaan, Kallenbach, & Rijhumal, 2010).

### The translated and adapted Sepedi version of the recalling sentences subtest of the clinical evaluation of language fundamentals-4 (CELF-4)

The sentences of the recalling sentences subtest were translated into Sepedi and then back-translated into English by the second author, who is proficient in both English and Sepedi. The back-translations were direct translations maintaining the Sepedi word order. The back translations were compared to the original English sentences to check that the number of clause structure elements (Subject, Verb, Object, Adverb, etc.) was maintained as described in the language assessment remediation and screening procedure (LARSP) (Crystal, Fletcher, & Garman, 1976). This procedure is recommended by Marinis and Armon-Lotem (2015) who developed the LITMUS-SRep tasks in different languages within COST Action IS0804.

To further ensure the validity of the Sepedi SR test, we made use of eight adult verifiers who spoke Sepedi as a first language, were residents of Sheshego or Polokwane and therefore from the same sociolinguistic environment as the learners (Thornton, 2015). The adult verifiers were asked to comment on the appropriateness of the Sepedi sentences and vocabulary items were changed if the majority of verifiers agreed on more appropriate terms.

## Procedures for data collection

The learners were assessed individually on both the English and Sepedi SR tests by the second author at their respective schools. In each school, half the learners were assessed in Sepedi first, followed by English and the other half in English first, followed by Sepedi. This was to counterbalance any order effects. The assessments took place in a quiet room during school hours but with minimal disruption to the academic programme.

Instructions to the learners were standardised and provided in the language of testing, that is, ‘please repeat these sentences after me’. Responses were recorded online by the researcher and one adult verifier by marking errors on pre-printed lists of sentences for each participant. Assessment took approximately 15 min – 20 min per learner for both SR tests. All items were administered and no ceiling procedure was used to discontinue item administration. In accordance with the administration manual, we implemented a graded scoring system for both the English and Sepedi tests. Learners were awarded three points for semantically and syntactically intact sentences, two points if there was one error in the sentence, one point for two to three errors and zero points for four or more errors. Any variation in the pronunciation of words because of articulation errors or accent was not considered as error.

## Reliability and validity

Validity of the SR measures has been addressed in their descriptions above. Test–retest reliability was addressed by testing a small sample (*n* = 5) in each group of learners twice. Reliability in scoring was addressed by ensuring 100% agreement between the researcher and adult verifier in scores awarded to each participant.

## Data analysis

The total raw score out of a possible 96 (32 sentences × three points for a perfect repetition) for each language was recorded for each participant on an Excel spreadsheet, containing an assigned number, the participant’s educational context, gender and age. The mean raw scores and standard deviations (SD) obtained by the participants in each language in each educational context were calculated.

Between-group comparisons were performed using independent sample *t*-tests to determine whether there were significant differences between the performance of learners in the three educational contexts on either the English or Sepedi SR tests. Within-group comparisons were performed using paired sample *t*-tests to determine whether there were significant differences between the learners’ performances in English and Sepedi within each educational setting (Howell, 2013).

According to the guidelines proposed by Rice (2009) and Bishop and McDonald (2009), learners whose scores were between 1 and 2 SD below the peer group means on the SR tests were identified as possibly language impaired. These authors suggest that children with SLI will generally score between 1 and 2 SD lower than their typically developing peers on most language measures.

### Ethical consideration

The Human Research Ethics Committee (Non-Medical) of the University of the Witwatersrand, Johannesburg, issued an ethical clearance certificate (Protocol No: H14/04/34) approving the study. Permission was obtained from the Limpopo Department of Education and from the respective school principals. As the participants were under the legal age of 18, informed consent was obtained from their parents and/or legal guardians and assent was obtained from those learners whose parents gave consent to their participation in the research. Anonymity was maintained by assigning a number to each participant.

Learners who were identified with possible language impairment were managed through appropriate mechanisms. The researchers notified the parents and/or guardians of the identified learners in writing and enclosed a referral letter citing the learners’ performance on the two SR measures and any scholastic difficulties identified by the grade teacher as reasons for referring these learners for further speech and language assessment at either Seshego or Polokwane Hospital.

## Results

The mean raw scores, SD and range of scores obtained by the learners in each educational context on the English and Sepedi SR tests are presented in Table 2. Raw scores have been converted to percentages for ease of interpretation.

**TABLE 2:** Mean raw scores, standard deviations and range of scores obtained by learners in each educational context on the English and Sepedi sentence repetition tests.

Educational context	Mean English raw score (Total possible = 96)		Mean Sepedi raw score (Total possible = 96)		Standard deviation		Score range	
	*n*	%	*n*	%	English	Sepedi	English	Sepedi
ELoLT	43.86	45.69	29.14	30.35	10.39	6.49	17–58	8–39
SLoLT-EAL	18.09	18.84	49.97	52.05	9.43	8.70	5–45	32–76
ELoLT -SAL	52.81	55.01	51.61	53.76	13.14	9.04	18–86	20–74

ELoLT, English language of learning and teaching; SLoLT-EAL, Sepedi language of learning and teaching-English additional language; ELoLT-SAL, English language of learning and teaching-Sepedi additional language.

### Between-group comparisons

With respect to the mean English SR test scores, learners in the ELoLT and ELoLT-SAL contexts obtained significantly higher scores (43.86 and 52.81, respectively) than those in the SLoLT-EAL context (18.09) (*t* = 11.10; *p* = 0.0000; *df* = 69) (*t* = 12.62; *p* = 0.0000; *df* = 64). The difference between the mean English scores in the ELoLT and ELoLT-SAL context was not significant (*t* = 3.36; *p* = 0. 0.3422; *df* = 65).

On the Sepedi SR test, learners in the ELoLT context obtained a significantly lower mean score (29.14) than the learners in both the SLoLT- EAL (*t* = 11.63; *p* = 0.0000; *df* = 69) and the ELoLT-SAL contexts (*t* = 11.98; *p* = 0.0000; *df* = 65), where the mean scores were 49.97 and 51.61 respectively. The difference between the means obtained by the ELoLT-SAL and SLoLT-EAL learners was not significant (*t* = 1.22; *p* = 45.5225; *df* = 64) on the Sepedi SR test.

### Within-group comparisons

The mean score on the English SR test (43.86) was significantly higher than on the Sepedi SR test (29.14) in the ELoLT group (*t* = 13.05; *p* = 0.0000; *df* = 35), whilst the mean score on the Sepedi SR test (49.97) was significantly higher than on the English SR test (18.09) in the SLoLT-EAL group (*t* = 25.04; *p* = 0.0000; *df* = 34).

The difference between the means on the English (52.81) and Sepedi (51.61) SR tests was not significant in the ELoLT-SAL group (*t* = 1.41; *p* = 33.8920; *df* = 30).

## Identification of learners with possible specific language impairment

As previously stated, learners whose scores were between 1 and 2 SD below the peer group means on the SR tests were identified as possibly language impaired, according to the guidelines proposed by Rice (2009) and Bishop and McDonald (2009).

The process of identifying learners with possible SLI in each context, using the means and SD, is illustrated in Table 3.

**TABLE 3:** Criteria for identification of learners with possible specific language impairment in each educational context.

Context	Language of SR test	Mean raw scores	Standard deviation	Raw score		Selection criteria
				1 SD below the mean	2 SD below the mean	
ELoLT	English	43.86	10.39	33. 37	23.08	Raw scores below 23 and 33
	Sepedi	29.14	6.49	22. 65	16.16	Raw scores below 16 and 22
SLoLT-EAL	English	18.09	9.43	8.65	-	Raw scores below 8
	Sepedi	49.97	8.70	41.27	32.57	Raw scores below 32 and 41
ELoLT -SAL	English	52.81	13.14	39.67	26.53	Raw scores below 26 and 39
	Sepedi	51.61	9.04	42.57	33.53	Raw scores below 33 and 42

SR, Sentence repetition; SD, Standard deviations; ELoLT, English language of learning and teaching; SLoLT-EAL, Sepedi language of learning and teaching-English additional language; ELoLT-SAL, English language of learning and teaching-Sepedi additional language.

Initially, 13 learners who scored between 1 and 2 SD below the peer group mean on either the English or the Sepedi SR tests were identified and their scores are reflected in Table 4. The scores of participants who scored 2 SD below the peer group mean are highlighted in yellow, whilst those scoring 1 SD below the mean are highlighted in blue on the table.

**TABLE 4:** Scores obtained by learners identified with specific language impairment in each educational context on the English and Sepedi sentence repetition tests.

Context	Participant	English SR score	SD below mean	Sepedi SR score	SD below mean
English LoLT	1	18†	2	31	N/A
	2	17†	2	16‡	1
	3	20†	2	8†	2
	4	21†	2	19‡	1
Sepedi LoLT-EAL	5	6‡	1	32‡	1
	6	12	N/A	33‡	1
	7	9	N/A	34‡	1
	8	6‡	1	35‡	1
	9	5‡	1	37‡	1
English LoLT-Sepedi Additional language	10	34‡	1	36‡	1
	11	31‡	1	39‡	1
	12	32‡	1	50	N/A
	13	18†	2	20†	2

† , 2 standard deviations below the mean;

‡ , 1 standard deviation below the mean.

SR, sentence repetition; SD, standard deviations; N/A, not applicable; LoLT, language of learning and teaching; EAL, English additional language.

## Discussion

The mean raw scores and percentages captured in Tables 2 and 3, suggest that best accuracy scores obtained by the Grade 3 learners in this study were between 50% and 55% in both languages. Although these scores may seem low and suggest that SR is not a simple task for these Grade 3 children, Marshall (2013), who conducted a study on the performance of Grade 2 EAL learners on the CELF-4 English SR measure, found that her participants obtained an even lower mean raw score of 32/96 (34%). In addition, the CELF-4 scoring manual reflects a normative mean raw score on the SR test for 8 year 8 month old monolingual children as between 50 and 55/96 (52% – 57%). The results achieved by the children in this study are thus in line with the normative data, which suggests that the results are valid.

However, the results of this study also confirm that bilingual children who speak the same languages are not a homogenous group and their SR abilities depend on their exposure to and experience in each language. The data highlight the importance of considering educational context in interpreting the results of language assessments and support the argument that establishing ‘bilingual norms’ (Armon-Lotem & Meir, 2016, p. 277) is not feasible.

The comparisons between the three contexts investigated in this study showed that participants obtained better scores in the LoLT, irrespective of whether this was English or Sepedi. The group who had English as an LoLT and were taught Sepedi as an additional language provide very informative results. Not only was the difference between their SR scores in English and Sepedi non-significant, they obtained a higher score in English (55.01%) than the English-LoLT (45.69%) group and a slightly higher score in Sepedi (53.76%) than the Sepedi LoLT group (52.05%). Although these differences were not statistically significant, they do support an additive approach to bilingualism where both languages are developed in an academic context. Their results also show that a bilingual approach can be achieved by teaching the home language as an additional language and that this leads to higher levels of bilingualism.

The English LoLT group obtained a significantly higher score (45.69%) in English than in Sepedi (30.35%) and a significantly lower score than the other two groups in Sepedi. Their poor performance in the home language suggests that these children may be experiencing a shift in language dominance to English and possible attrition of the home language, which is disconcerting given the imperative to promote bilingualism and maintain the development of all South African languages (LiEP, 1997). In schools where English is the LoLT, teachers and parents should make every attempt to use the home language, not only for daily communication but also as a language of learning. This can be done by relating what is taught in English to the home language.

The Sepedi LoLT group obtained a significantly higher score in Sepedi (52.05%) than in English (18.84%), which is their additional language and in which they obtained a significantly lower score than the other two groups. Given that these learners are to transition to English as the LoLT in grade 4, this is a worrying finding as it suggests that they have not mastered English sufficiently to use it for academic purposes. The fact that their Sepedi SR score (52.05%) is very similar to that of the group who were taught Sepedi as an additional language (53.67%), would counteract any argument that their language skills may be negatively affected because they were from a lower socio-economic group than the other two groups. It may also suggest that SR is not as sensitive to socio-economic differences as other assessment measures, such as vocabulary tests.

Nonetheless, the implications for this group are either that much more needs to be done to develop their English proficiency or the home language needs to be used as the LoLT beyond Grade 3 in this context.

This study has demonstrated that SR, a relatively quick and easy task to administer, can be used as a screening tool to identify bilingual children with possible SLI by comparing them to a peer group. As shown in Table 4, 13 learners were initially identified with possible SLI. Of these, one learner (P1) in the English LoLT context could be excluded, because although he scored 2 SD below the mean in English, he did not score below the mean in his home language, Sepedi. Similarly, P12 in the ELoLT-SAL context, who scored 1 SD below the mean on the English SR test but not below the mean on the Sepedi SR test, could be excluded. Both these participants seemed to be in the process of acquiring English, but have stronger language skills in their home language. However, if their assessments were based on English only, they would have been over-identified as language impaired. Therefore, the importance of assessing bilingual children in both languages is underlined. These two learners merely need support in English as the LoLT.

Two learners (P3 and P13) obtained scores 2 SD below the peer group means in both English and Sepedi and may be considered severely impaired. Two learners (P2 and P4) in the English LoLT group obtained English SR scores 2 SD below the mean and Sepedi SR scores 1 SD below the mean, suggesting slightly better home language skills and significant difficulty acquiring the LoLT. These learners would be at a risk of academic difficulty but may benefit from support in English and their home language. These results also show that SR scores can estimate the degree of difficulty in each language.

Of the five learners identified in the Sepedi LoLT –EAL context, two (P6 and P7) should be followed up for further assessment because they scored 1 SD below the peer group mean on the Sepedi SR test, although their English scores were not below the 1 SD cut-off score. As Sepedi is their home language and LoLT, they may well be at risk for SLI.

Three learners in the Sepedi LoLT- EAL context (P5, P8 and P9) and two learners in the English LoLT- SAL context (P10 and P11) scored 1 SD below the peer group mean on both the English and Sepedi SR tests and may be confidently identified with possible SLI.

## Conclusion

The findings of this study suggest that speech-language therapists working in educational contexts can use SR tasks as screening tools to identify bilingual children with language impairments by comparing them to peer groups. This population-based strategy is likely to yield far more accurate and valid identification than other measures. Of course, this would require the development of SR tests in all South African languages, but clinicians and researchers are encouraged to take up this challenge, particularly because SR has strong theoretical foundations as a comprehensive language assessment tool. The SR tests used in this study were clearly sensitive to language practices in different educational contexts, which enhance their validity. In this regard, the study also highlights a number of important implications for language-in-education practices and confirms that a bilingual approach in which both English and the home language are fully developed as academic languages leads to better language outcomes. Although a bilingual approach may be challenging in schools where home languages are highly heterogeneous, ways of enhancing the development of learners’ first languages should be sought as far as possible.
